# mGluR5 positive modulators both potentiate activation and restore inhibition in NMDA receptors by PKC dependent pathway

**DOI:** 10.1186/1423-0127-18-19

**Published:** 2011-02-22

**Authors:** Hwei-Hsien Chen, Pei-Fei Liao, Ming-Huan Chan

**Affiliations:** 1 Institute of Pharmacology and Toxicology, Tzu Chi University, Hualien, Taiwan

## Abstract

**Background:**

In order to understand the interaction between the metabotropic glutamate subtype 5 (mGluR5) and N-methyl-D-aspartate (NMDA) receptors, the influence of mGluR5 positive modulators in the inhibition of NMDA receptors by the noncompetitive antagonist ketamine, the competitive antagonist D-APV and the selective NR2B inhibitor ifenprodil was investigated.

**Methods:**

This study used the multi-electrode dish (MED) system to observe field potentials in hippocampal slices of mice.

**Results:**

Data showed that the mGluR5 agonist (RS)-2-chloro-5-hydroxyphenylglycine (CHPG), as well as the positive allosteric modulators 3-cyano-*N*-(1,3-diphenyl-1*H*-pyrazol-5-yl) benzamide (CDPPB) and 3,3'-difluorobenzaldazine (DFB) alone did not alter the basal field potentials, but enhanced the amplitude of field potentials induced by NMDA. The inhibitory action of ketamine on NMDA-induced response was reversed by CHPG, DFB, and CDPPB, whereas the blockade of NMDA receptor by D-APV was restored by CHPG and CDPPB, but not by DFB. Alternatively, activation of NMDA receptors prior to the application of mGluR5 modulators, CHPG was able to enhance NMDA-induced field potentials and reverse the suppressive effect of ketamine and D-APV, but not ifenprodil. In addition, chelerythrine chloride (CTC), a protein kinase C (PKC) inhibitor, blocked the regulation of mGluR5 positive modulators in enhancing NMDA receptor activation and recovering NMDA receptor inhibition. The PKC activator (PMA) mimicked the effects of mGluR5 positive modulators on enhancing NMDA receptor activation and reversing NMDA antagonist-evoked NMDA receptor suppression.

**Conclusion:**

Our results demonstrate that the PKC-dependent pathway may be involved in the positive modulation of mGluR5 resulting in potentiating NMDA receptor activation and reversing NMDA receptor suppression induced by NMDA antagonists.

## Introduction

Glutamate is a well-known excitatory neurotransmitter in the mammalian central nervous system (CNS) and plays an important role by acting through two distinct types of receptors, the ion-channel associated (ionotropic) and G-protein-coupled (metabotropic) receptors [1]. Ionotropic glutamate receptors (iGluRs) that mediate fast excitatory synaptic transmission are ion channels permeable to cations and are classified as α-amino-3-hydroxy-5-methyl-4-isoazolepropionic acid (AMPA), kainite, and N-methyl-D-aspartate (NMDA) receptors based on agonist preference. Metabotropic glutamate receptors (mGluRs) are members of G-protein-coupled receptor (GPCR) and influence a variety of intracellular second messenger systems that modulate neuronal excitability, synaptic plasticity, and neurodegeneration. mGluRs are involved in physiological and pathophysiological processes, including development, learning and memory, pain, ischemia, stroke, epileptic seizures, schizophrenia, as well as chronic neurodegenerative diseases [2]. Eight mGluR subtypes have been identified and divided into three subgroups based on sequence homology, signal transduction pathways, and pharmacology [3]. They are Group I (mGluR1 and mGluR5), Group II (mGluR2 and mGluR3), and Group III (mGluR4, mGluR6, mGluR7, and mGluR8). Among these three groups of mGluRs, Group I mGluRs (mGluR1/5) have drawn the most attention because of their wide distribution in CNS and active regulation of multiple neuronal signaling. Stimulation of these receptors by agonists increases hydrolysis of membrane phosphoinositide (PI) via activated phospholipase C, leading to formation of diacylglycerol (DAG), which activates protein kinase C (PKC) and inositol-1,4,5-trisphosphate (IP3), which induces calcium release from intracellular stores and then stimulates PKC [4,5]. Furthermore, the alteration of PKC and intracellular calcium signals could modulate various metabotropic functions.

Interactions between mGluRs and NMDA receptors have been described [6]. Activation of NMDA receptors provides a facilitatory regulation of mGluR5 responses [7,8]. Conversely, mGluR5 is physically connected with NMDA receptors and their stimulation positively modulates the function of NMDAergic synapse in several brain regions [9,10]. Recent behavioral studies also demonstrated that mGluR5 antagonists augment the noncompetitive NMDA receptor antagonists, PCP or MK-801, induced responses such as locomotor hyperactivity, impairment of prepulse inhibition [11,12], and cognitive deficits [13]. Previously, we have also reported that the mGluR5 agonist (RS)-2-chloro-5-hydroxyphenylglycine (CHPG), and antagonist 2-methyl-6-(phenylethyl)-pyridine (MPEP) may respectively reduce and enhance the ketamine anesthesia [14]. Furthermore, the mGluR5 positive modulators attenuate ketamine-induced behavioral responses [15]. Accordingly, it is anticipated that mGluR5 positive modulators are capable of reversing the suppression of NMDA receptors in response to noncompetitive NMDA receptor antagonists. However, the interactions of mGluR5 positive modulators with NMDA receptor antagonists remain unclear.

In the present study, we set out to delineate the interacting effect of mGluR5 and NMDA receptor antagonists on NMDA channel activity. Recently, a novel class of potent positive allosteric modulators of mGluR5 has been discovered [16-19]. For example, 3-cyano-N-(1,3-diphenyl-1H-pyrazol-5-yl)benzamide (CDPPB) and 3,3'-difluorobenzaldazine (DFB) have no agonist activity but potentiate threshold responses to glutamate, quisqualate and (S)-3,5-dihydroxyphenylglycine. Therefore, our experiments determined whether the mGluR5 agonist, CHPG, and the positive allosteric mGluR5 modulators, DFB and CDPPB, could potentiate NMDA receptor activation and/or restore NMDA receptor suppression induced by ketamine, a noncompetitive NMDA receptor antagonist, D-APV, a selective NMDA receptor antagonist, and ifenprodil, a NR2B selective NMDA receptor antagonist, via measuring the field potentials in hippocampal slices of mice.

## Materials and methods

### Animal and Materials

Male NMRI mice (8-9 weeks, 33-40 g) were supplied from the Laboratory Animal Center of Tzu Chi University (Hualien, Taiwan) and were housed 4 to 5 per cage in a 12 hr light/dark cycle with ad libitum access to water and food. The experimental protocol was approved by the Tzu Chi University Review Committee for the Use of Animals.

Glycine and potassium chloride were purchased from J.T. Baker (Mallinckrodt Baker, Inc, Kentucky, USA). RS-2-chloro-5-hydrophonovaleric acid (CHPG), chelerythrine chloride (CTC), 3, 3'-difluorobenzaldazine (DFB), phorbol 12-myristate 13-acetate (PMA), and tetrodotoxin (TTX) were purchased from Tocris (Northpoint Forth Way Avonmouth, UK). D-2-amino-5-phosphonovaleric acid (D-APV), ketamine, N-methyl-D-aspartic acid (NMDA), 4 α-phorbol 12, 13-didecanoate (4α-PDD) and other chemicals were obtained from Sigma (St Louis, MO, USA). For the preparation of stock solution, CHPG was initially dissolved in 0.5 N NaOH and then neutralized by 0.5 N HCl. DFB was dissolved in DMSO, whereas ketamine was dissolved in saline. Then the individual reagents were diluted in an artificial cerebrospinal fluid (ACSF) containing (in mM) NaCl (120), KCl (3.5), CaCl_2 _(2.5), MgCl_2 _(1.2), NaHCO_3 _(25), NaH_2_PO_4 _(1.2), and D-glucose (11.5) at pH 7.4.

### Preparation of multielectrode array

The preparation of the multi-electrode dish (MED; Panasonic, Japan) has been described previously by Oka et al. (1999). The MED probe is an array of 64 planar microelectrodes, where each microelectrode has a size of 50 × 50 μm and is arranged in an 8 × 8 pattern. The interpolar distance in this type of probe (MED-P515A) is 150 μm. For sufficient adhesion of the hippocampal slice to the MED probe, the surface of probe was treated with 0.1% polyethylenimine or collagen in 25 mM borate buffer for 8 hr at room temperature. Then the probe surface was rinsed three times with distilled water for future experiments.

### Preparation of hippocampal slices

The NMRI mice were sacrificed by decapitation after anesthesia, and the whole brain was carefully removed. The brain was then immediately soaked in ice-cold and oxygenated ACSF. Appropriate portions of the brain were trimmed and placed on the ice-cold stage of a vibrating tissue slicer, whereas the stage was filled with oxygenated ACSF. Each slice (300 μm) was gently taken off the blade with a paint brush, trimmed, and immediately re-soaked in ACSF under 95% O_2_/5% CO_2 _bubbling for 90 min at room temperature. Then the hippocampal slice between CA3 and CA1 was placed on the center of the coated MED probe and positioned to cover the 8 × 8 microelectrode array. After positioning the hippocampal slice on MED probe, the ACSF was applied to the slice up to an interface level.

### Electrophysiological recordings

For electrophysiological recordings, the MED probe containing the hippocampal slice was placed in a small incubator which was superfused with ACSF in 5% CO_2_/95% O_2 _at 34°C and connected to the stimulation/recording component of MED8. The spontaneous field potential or chemical evoked field potential at all 64 sites in the 64 multi-electrode probe was recorded simultaneously with the multi-channel recording system (Panasonic; MED8 system) at a 20 kHz sampling rate. The electrodes in the stratum radiatum of field CA1 were selected as the recording electrodes. The recording of field potentials was first carried out in the absence of any chemical and electrical stimulation to establish a baseline. In order to prevent the sodium channel mediated spontaneous components, all the following experiments were performed with 0.3 μM TTX. For drug treatment purpose, ACSF containing appropriate concentrations of various drugs were applied.

### Statistical analyses

The recording channels for analysis were selected among the electrodes located in the stratum radiatum of field CA1. The maximum amplitudes of field potentials were measured. All data are expressed as mean ± S.E.M. Statistical significance of the difference between groups was determined by one-way ANOVA followed by a Student-Newman-Keuls post-hoc test. P < 0.05 was considered statistically significant.

## Results

### NMDA-induced potentials inhibited by ketamine, D-APV, and ifenprodil

**Figure 1:**
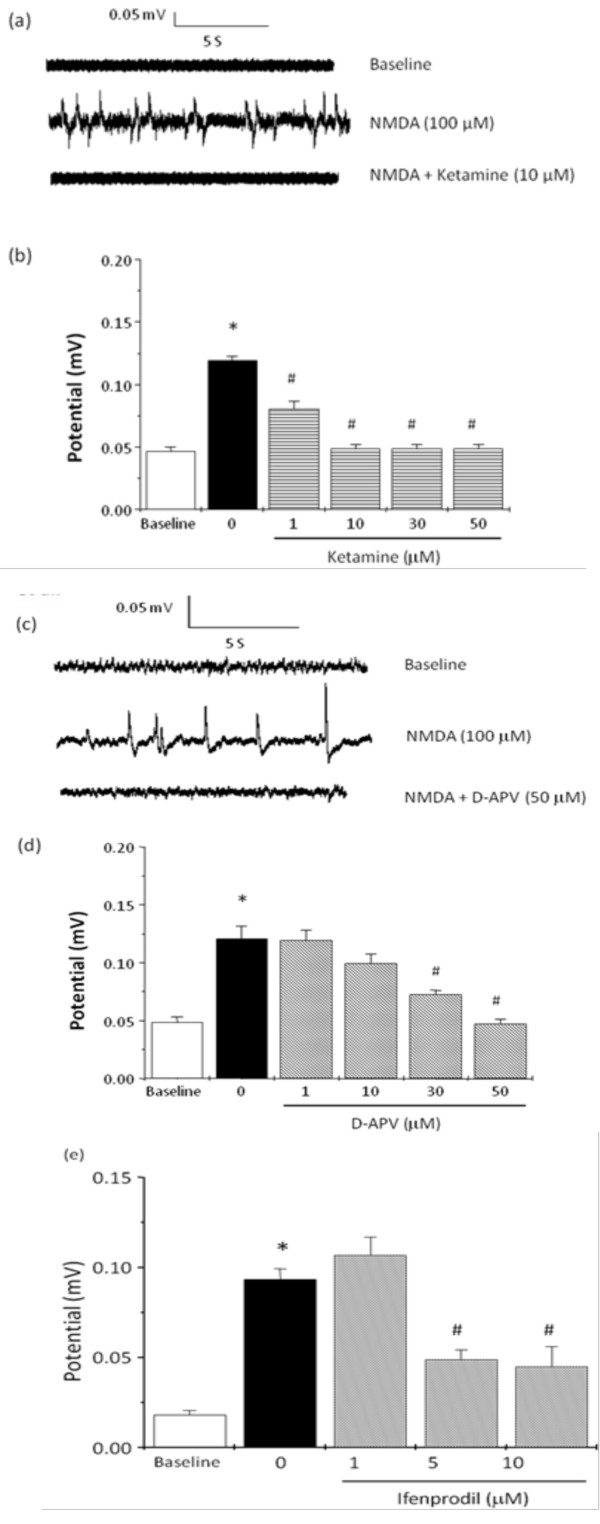
**Inhibitory effects of ketamine, D-APV and ifenprodil on NMDA-induced potentials in hippocampal slices of mice**. A representative recordings show the field potentials induced by NMDA (100 μM) and co-application of NMDA with ketamine (a, 10 μM) or D-APV (c, 50 μM). (b, d, e) Histograms represent the average amplitude of field potentials during superfusion of hippocampal slices with NMDA and co-application of NMDA together with ketamine, D-APV, or ifenprodil at the concentrations of 1-50 μM. All values are expressed as the mean ± S.E.M (n = 6). Data were analyzed by one-way ANOVA followed by a Student-Newman-Keuls post-hoc test. *P < 0.05 as compared with the baseline. ^#^P < 0.05 as compared with the NMDA groups and treated with NMDA alone.

Figure 1 illustrates the representative recordings of field potentials in hippocampal slices of mouse brains. The basal spontaneous potential in an individual hippocampal slice was initially recorded for 5 min, and then NMDA (100 μM) was applied to stimulate field potentials, followed by co-administration with NMDA receptor antagonists such as ketamine, D-APV, or ifenprodil. The present data demonstrated that the baseline activity of field potential was of low voltage under TTX treatment in the mouse hippocampus. Infusion of NMDA (100 μM) into the hippocampal slice was observed to significantly evoke field potentials. These NMDA-induced field potentials were blocked by NMDA receptor inhibitors ketamine (1-50 μM), D-APV (1-50 μM), and ifenprodil (1-10 μM) in a concentration-dependent manner (Figure 1). Moreover, ketamine at the concentration of 10 μM and D-APV at 50 μM attenuated the amplitude of filed potentials induced by NMDA, which approached to the baseline level.

### Effects of mGluR5 modulators on NMDA receptor activation and suppression

**Figure 2:**
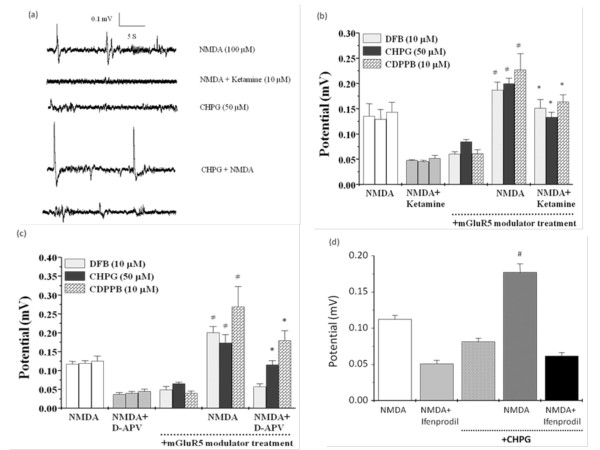
**Effects of mGluR5 modulators on NMDA-induced field potentials and the NMDA receptor blockade by ketamine, D-APV, or ifenprodil in hippocampal slices**. (a) A representative recording showing co-application of CHPG (50 μM) enhanced NMDA (100 μM)-induced potentials and prevented ketamine (10 μM)-evoked suppression on NMDA receptors. Summary data showing the average amplitude of field potentials induced by NMDA with and without ketamine (b, 10 μM), D-APV (c, 50 μM), or ifenprodil (d, 5 μM) as well as in the co-application of mGluR5 modulators, DFB (10 μM), CHPG (50 μM), or CDPPB (10 μM). All values are expressed as the mean ± S.E.M. (n = 5-7). Data were analyzed by one-way ANOVA followed by a Student-Newman-Keuls post-hoc test. ^#^p < 0.05 as compared with the NMDA groups. *p < 0.05 as compared with the NMDA plus ketamine or D-APV groups.

The mGluR5 modulators including DFB, CHPG, and CDPPB were used to test their regulation on NMDA receptor activation and suppression. In the following experiments, we also initially recorded the field potentials induced by NMDA (100 μM) and then by co-application of NMDA with ketamine, D-APV, or ifenprodil at the respective concentration of 10 μM, 50 μM, or 5 μM, which was utilized to elicit the appropriate inhibition on NMDA-induced field potentials. After 10 min of washout, hippocampal slices were exposed to the mGluR5 modulator, and then mGluR5 modulator combined with NMDA for 5 min, followed by co-application of mGluR5 modulator, NMDA, and NMDA receptor antagonist ketamine, D-APV, or ifenprodil. Here we observed that DFB (10 μM), CHPG (50 μM), or CDPPB (10 μM) alone did not alter the basal field potentials in mice hippocampus (Figure 2). Importantly, pretreatment of slices with DFB, CHPG, or CDPPB followed by an NMDA application, the amplitude of NMDA-induced field potentials was significantly enhanced. Furthermore, both CHPG and CDPPB reversed the blockade of ketamine and D-APV on NMDA-induced field potentials (Figure 2). DFB also significantly reversed the inhibitory responses elicited by ketamine, but not by D-APV (Figure 2c). However, CHPG did not influence ifenprodil-elicited suppression on NMDA receptor activation (Figure 2d)

**Figure 3:**
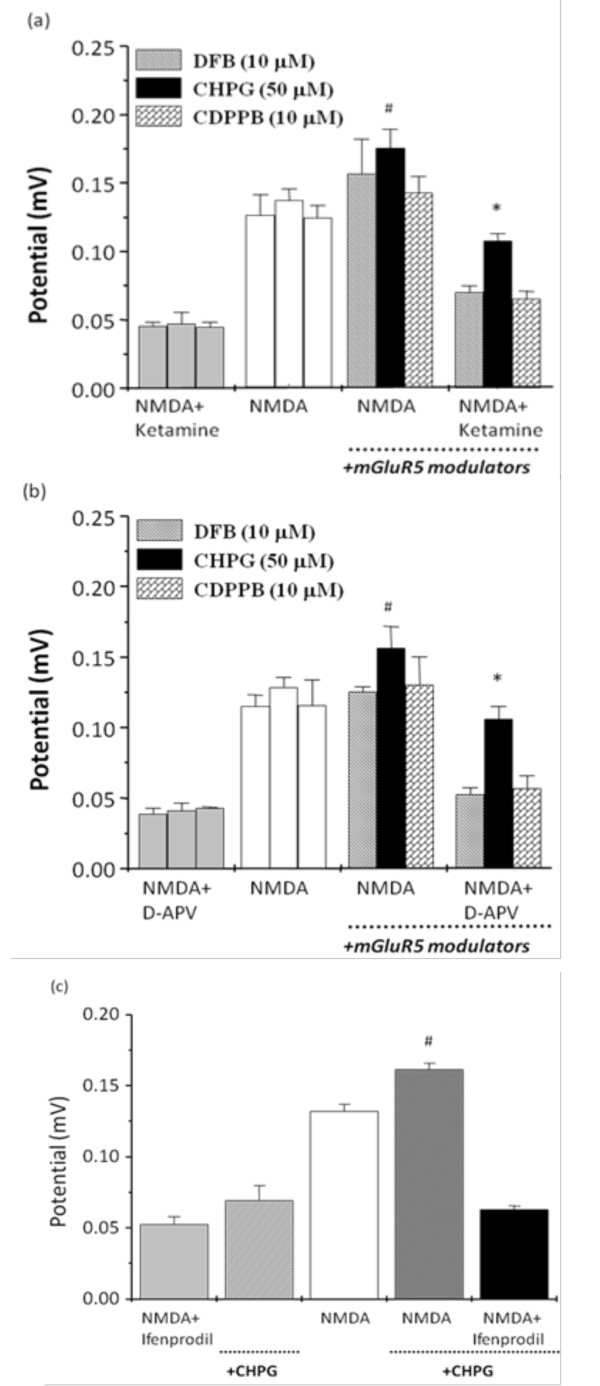
**Pretreatment of NMDA alters the regulation of mGluR5 modulators on the potentiation of NMDA activation and prevention of NMDA blockade**. The hippocampal slices were initially exposed to NMDA with ketamine (a, 10 μM), D-APV (b, 50 μM), or ifenprodil (c, 5 μM). After washout, the NMDA was re-exposed to the mGluR5 modulator, DFB (10 μM), CHPG (50 μM), or CDPPB (10 μM) was co-applied with NMDA, followed by a co-application of NMDA, the mGluR5 modulator, and ketamine, D-APV, or ifenprodil. Summary data showing the average amplitude of field potentials induced by NMDA with and without ketamine, D-APV, or ifenprodil as well as in the co-application of mGluR5 modulators, DFB (10 μM), CHPG (50 μM), or CDPPB (10 μM). All values are expressed as the mean ± S.E.M. (n = 5-7). Data were statistically analyzed by one-way ANOVA followed by a Student-Newman-Keuls post-hoc test. ^#^p < 0.05 as compared with the NMDA groups. *p < 0.05 as compared with the NMDA plus ketamine or D-APV groups.

When the exposure of NMDA to hippocampal slices was conducted prior to co-application of positive mGluR5 modulators combined with NMDA, it was observed that the NMDA-induced field potential was particularly potentiated by CHPG, but not by DFB and CDPPB. The inhibitory effects of ketamine and D-APV on NMDA receptors were only significantly reversed by CHPG, but not by DFB and CDPPB (Figure 3a, b). In contrast, the inhibitory effect of ifenprodil on NMDA receptor activation was not reversed by CHPG (Figure 3c).

### PKC dependent pathway

**Figure 4:**
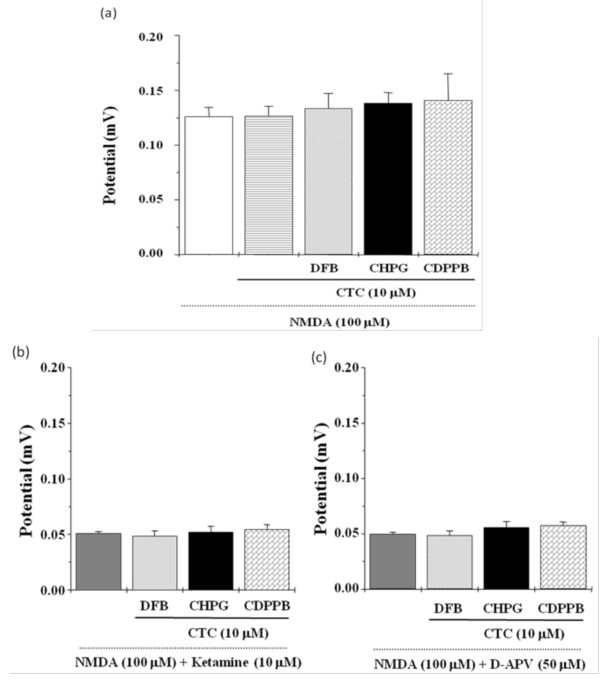
**PKC inhibitor influences the effects of mGluR5 modulators on NMDA receptor activation and suppression**. Pretreatment of hippocampal slices with chelerythrine (10 μM), a PKC inhibitor, inhibited the enhancing effects of DFB (10 μM), CHPG (50 μM), and CDPPB (10 μM) on NMDA-induced field potentials (a) and reversed the attenuating effects of mGluR5 modulators on ketamine (b, 10 μM) or D-APV (c, 50 μM)-evoked NMDA receptor suppression. All values are expressed as the mean ± S.E.M. (n = 5). Data were statistically analyzed by one-way ANOVA followed by a Student-Newman-Keuls post-hoc test.

**Figure 5:**
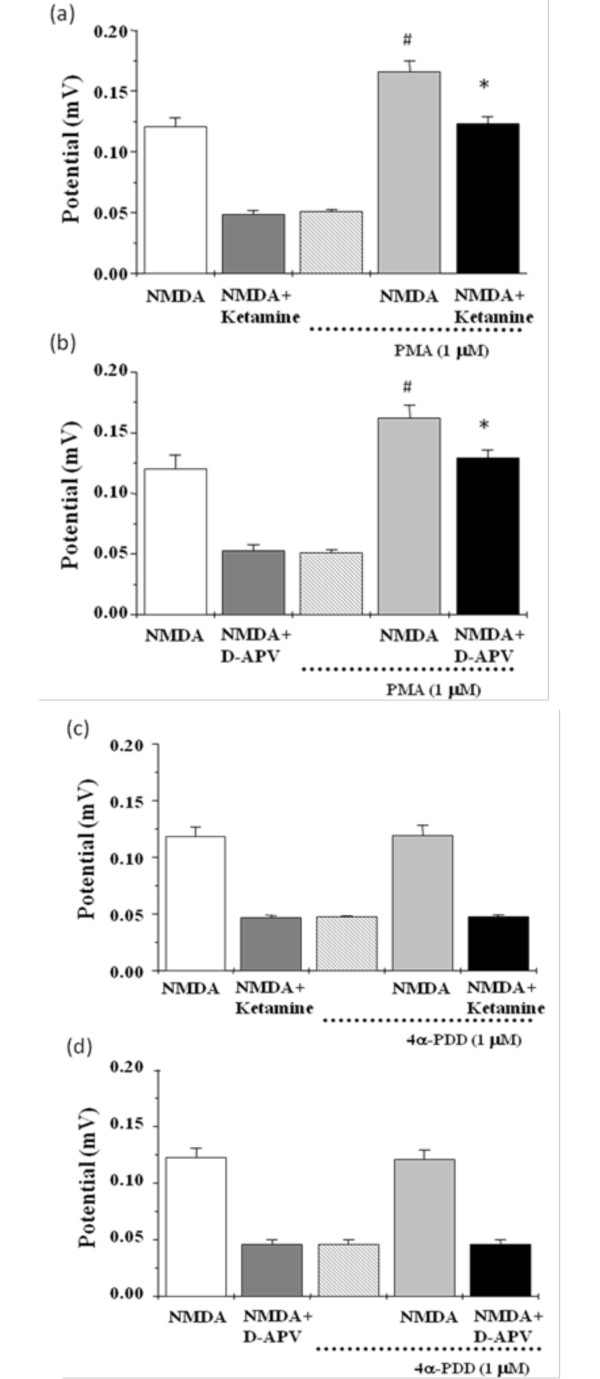
**Effects of phorbol ester on NMDA receptor activation and suppression**. Pretreatment of hippocampal slices with PMA (1 μM, a PKC activator) potentiating NMDA-induced field potentials (a, b) and prevented ketamine (a, 10 μM)- or D-APV (b, 50 μM)-evoked NMDA receptor blockade. Pretreatment of hippocampal slices with 4α-PDD (1 μM, inactive phorbol ester) did not affect NMDA-elicited field potentials (c, d) and ketamine (c, 10 μM)- or D-APV (d, 50 μM)-induced NMDA receptor blockade. All values are expressed as the mean ± S.E.M. (n = 6). Data were statistically analyzed by one-way ANOVA followed by a Student-Newman-Keuls post-hoc test. ^#^p < 0.05 as compared with the NMDA groups. *p < 0.05 as compared with the NMDA plus ketamine or D-APV groups.

The following experiments were to determine whether the influence of NMDA receptor activation and suppression by positive mGluR5 modulators was involved in the protein kinase C (PKC) dependent pathway. Pretreatment with chelerythrine (CTC, 10 μM), a PKC blocker, was found to inhibit the potentiation of DFB, CHPG, and CDPPB on NMDA-induced field potentials (Figure 4a). Chelerythrine also blocked the reversing effects of DFB, CHPG, and CDPPB on ketamine- and D-APV-evoked NMDA receptor suppression (Figure 4b, c). Importantly, the field potential induced by NMDA was also enhanced by PMA (1 μM), the PKC activator, but not by 4α-PDD (1 μM), inactive phorbol esters (Figure 5). Furthermore, the inhibitory effects of ketamine and D-APV on NMDA receptor activation were reversed by PMA (1 μM), but not by 4α-PDD (1 μM) (Figure 5).

## Discussion

In the present study, we examined the effects of the mGluR5 orthosteric agonist, CHPG, and the mGluR5 positive allosteric modulators, DFB and CDPPB, on hippocampal filed potentials induced by NMDA receptor activation and inhibited by NMDA receptor antagonists such as ketamine, D-APV, and ifenprodil. Our results demonstrated that pretreatment with CHPG, DFB, and CDPPB produced a predominate augmentation on field potentials induced by NMDA in the hippocampal slice, although they alone did not affect the basal potential. These mGluR5 modulators also reserved the inhibitory actions of ketamine and D-APV on NMDA-elicited responses. As NMDA was pretreated before co-application of mGluR5 modulators, the potentiation of NMDA receptor activation and restoration of NMDA receptor blockade were regulated by CHPG, but not by DFB and CDPPB. Furthermore, the mGluR5-mediated amplification of NMDA-induced potentials and restoration of NMDA receptor blockade were blocked by the PKC inhibitor, Suggesting that the cellular modulation of NMDA receptor by mGluR5 may involve in PKC-dependent pathway. Therefore, our results indicated that these positive mGluR5 modulators could be effective in attenuating the hippocampal abnormalities that result from NMDA receptor hypofunction.

The potentiating actions of CHPG, DFB, and CDPPB on NMDA-induced field potentials are similar to previous reports, which demonstrated that the selective mGluR5 agonists produced enhancement of NMDA-mediated responses in rat hippocampal slices [20], rat subthalamic nucleus slices [9], and mouse striatal medium spiny neurons [10]. However, DFB, CDPPB, and even the selective mGluR5 agonist CHPG, when administrated alone, did not influence the basal field potentials in the hippocampal slices. Consistently, CHPG did not affect the field excitatory postsynaptic potential (fEPSP) in the CA1 area of rat hippocampal slices [21] and the ventral root potential in the rat spinal cord [22]. Furthermore, CHPG did not alter the ratio of fEPSP responses, indicating that CHPG may be unlikely to induce a presynaptic release of glutamate. When the concentration has reached higher than 1 mM, CHPG can elicit a reduction in the fEPSP slope [21], suggesting that mGluR5 activation may act on synaptic transmission through an increase in endogenous glutamate neurotransmission on NMDA receptors, as reported to occur in the striatum[23] and periaqueductal grey [24]. It appears that CHPG, DFB, and CDPPB at the concentrations used under this experimental condition mainly act postsynaptically and do not influence the glutamate neurotransmission in hippocampus, since the selective concentrations for these mGluR5 positive modulators (10-50 μM) were much lower than the concentration that elicits glutamate release.

The mGluR5 positive allosteric modulator CDPPB has been recently reported to reverse the effects of the noncompetitive NMDA antagonist MK801 on rat cortical neuronal firing [25]. In agreement with this previous report, our present study also demonstrated that positive modulation of mGluR5 restored the inhibitory effects of the NMDA receptor antagonist ketamine and D-APV on mouse hippocampal field potentials activated by NMDA. The above electrophysiological evidence may further reveal our recent findings that mGluR5 positive modulators attenuate ketamine-induced behavioral responses [15]. Alternatively, the mGluR5 antagonists can potentiate the neuronal firing evoked by NMDA receptor antagonists in rat cortical neurons [26]. In line with the potentiating actions of mGluR5 antagonists on the noncompetitive NMDA receptor antagonists-induced responses such as locomotor hyperactivity, prepulse inhibition [11,12] and cognitive deficits [13,27], these animal behavioral studies also reveal that activation of mGluR5 could ameliorate the behavioral abnormalities associated with NMDA receptor deficiency. Therefore, modulation of mGluR5 may provide a novel approach for the development of therapeutic agents to treat CNS impairment induced by NMDA receptor dysfunction.

Activation of mGluR5 has been demonstrated to facilitate NMDA receptor function [10,20] and reverse the effects of NMDA antagonist induced responses [15,25]. CHPG, directly binding to glutamate binding site, and DFB as well as CDPPB, binding to the heptahelical transmembrane domain of mGluR5, increase the intrinsic efficacy of the endogenous glutamate to activate mGluR5, which results in enhancement of NMDA receptor function and reversion of the NMDA receptor obstruction elicited by ketamine and D-APV. However, CHPG did not improve the inhibitory action of ifenprodil, a NR2B selective NMDA receptor antagonist [28], on NMDA receptor activation. It suggests that the regulation of NMDA receptor by mGluR5 may not involve the NMDA receptor subunit NR2B. Interestingly, the sensitivities of NMDA receptor activation and suppression in response to the mGluR5 agonist CHPG and the allosteric modulators DFB and CDPPB were remarkably distinct when the NMDA receptor was activated prior to the introduction of mGluR5. It is important to point out that with pretreatment of NMDA before mGluR5 activation, the potentiation of the NMDA receptor and restoration of receptor barrier were only regulated by CHPG, but not by DFB and CDPPB. These findings indicate that NMDA receptor activation may change the sensitivity of mGluR5 for agonists and allosteric modulators, since stimulation of NMDA receptor has been reported to induce phosphorylation of mGluR5 and activation of protein phosphatase [7,29]. It is possible that mGluR5 in this state is insensitive to allosteric modulators.

Two distinct signaling pathways for the potentiation of NMDA responses by mGluRs have been presented, one PKC-dependent pathway [30,31] and another PKC-independent process [32,33]. Our results showed that mGluR5 signals sent via PKC to enhance NMDA-mediated responses and restore the obstruction of NMDA receptor by specific antagonists, since the PKC inhibitor blunted mGluR5 positive modulators mediated NMDA potentiation and restoration of NMDA suppression. Furthermore, PKC activator has the similar effects of mGluR5 positive modulators through enhancing NMDA receptor activation and reversing the NMDA antagonist-evoked NMDA receptor suppression. The molecular interactions that mediate the actions of mGluR5 on NMDA receptors have been evidenced by the agonist-elicited increase in the phosphorylation of two serine residues (serine 896 and serine 897) of NR1 subunit of NMDA receptors [34]. Positive allosteric modulators also potentiate this response to a subthreshold concentration of agonist [35]. It is not known, however, whether phosphorylation of the NR1 receptors could reduce the efficacy of noncompetitive NMDA receptor antagonists, such as ketamine and D-APV. Further studies are needed to determine whether mGluR5 positive modulators influence the NMDA receptor activation and suppression via modification of the phosphorylation of NR1 subunit of NMDA receptors.

In accordance with previous evidence showing that mGluR5 positive modulators attenuate NMDA antagonist-evoked behavioral responses, our present data provide electrophysiological evidence that mGluR5 have modulatory effects on NMDA receptor activation and suppression, which are reversed by the PKC inhibitor. These findings suggest that the regulatory role of mGluR5 on NMDA receptor is involved in the PKC dependent pathway and support the notion that positive mGluR5 modulation is a potential therapeutic strategy in the treatment of NMDA receptor hypofunction such as schizophrenia.

## Competing interests

The authors declare that they have no competing interests.

## Authors' contributions

HHC and PFL contributed equally to this work. HHC participated in the design of the study and sequence alignment, performed the statistical analysis, and drafted the manuscript. PFL carried out the preparation of the hippocampal slices and performed the electrophysiological recordings. MHC conceived of the study and helped to draft the manuscript. All authors read and approved the final manuscript.
